# Health system gaps in cardiovascular disease prevention and management in Nepal

**DOI:** 10.1186/s12913-021-06681-0

**Published:** 2021-07-05

**Authors:** Archana Shrestha, Rashmi Maharjan, Biraj Man Karmacharya, Swornim Bajracharya, Niharika Jha, Soniya Shrestha, Anu Aryal, Phanindra Prasad Baral, Rajendra Dev Bhatt, Sanju Bhattarai, Durga Bista, David Citrin, Meghnath Dhimal, Annette L. Fitzpatrick, Anjani Kumar Jha, Robin Man Karmacharya, Sushmita Mali, Tamanna Neupane, Natalia Oli, Rajan Pandit, Surya Bahadur Parajuli, Pranil Man Singh Pradhan, Dipanker Prajapati, Manita Pyakurel, Prajjwal Pyakurel, Binuka Kulung Rai, Bhim Prasad Sapkota, Sujata Sapkota, Abha Shrestha, Anmol Purna Shrestha, Rajeev Shrestha, Guna Nidhi Sharma, Sumitra Sharma, Donna Spiegelman, Punya Shori Suwal, Bobby Thapa, Abhinav Vaidya, Dong Xu, Lijing L. Yan, Rajendra Koju

**Affiliations:** 1 Department of Public Health, Kathmandu University School of Medical Sciences, Dhulikhel, Kavre Nepal; 2 Department of Chronic Disease Epidemiology, Yale School of Public Health, New Haven, USA; 3 Institute for Implementation Science and Health, Kathmandu, Nepal; 4 Department of Community Programs, Dhulikhel Hospital Kathmandu University Hospital, Dhulikhel, Kavre Nepal; 5 Department of Nursing and Midwifery, Kathmandu University School of Medical Sciences, Dhulikhel, Kavre Nepal; 6 Nyaya Health Nepal, Kathmandu, Nepal; 7 Department of Health Services, Non Communicable Diseases and Mental Health Section, Epidemiology and Disease Control Division, Ministry of Health and Population, Government of Nepal, Kathmandu, Nepal; 8 Department of Biochemistry, Dhulikhel Hospital Kathmandu University Hospital, Dhulikhel, Kavre Nepal; 9 Faculty of Medical Sciences, School of Health Sciences, Wuhan University, Wuhan, China; 10 Department of Pharmacy, Kathmandu University, Dhulikhel, Kavre Nepal; 11 Possible, New York, NY USA; 12 Department of Global Health, University of Washington, Seattle, WA USA; 13 Department of Anthropology, University of Washington, Seattle, WA USA; 14 Medic, Seattle, WA USA; 15 Icahn School of Medicine at Mount Sinai, Arnhold Institute for Global Health, New York, NY USA; 16 Nepal Health Research Council, Ramshah Path, Kathmandu, Nepal; 17 Departments of Family Medicine, Epidemiology, and Global Health, University of Washington, Seattle, USA; 18 Department of Surgery (Cardio Thoracic and Vascular unit), Dhulikhel Hospital Kathmandu University Hospital, Dhulikhel, Kavre Nepal; 19 Department of Community Medicine, Kathmandu Medical College and Teaching Hospital, Kathmandu, Nepal; 20 Department of Physiology, Nepal Medical College and Teaching Hospital, Attarkhel, Kathmandu, Nepal; 21 Department of Community Medicine, Birat Medical College and Teaching Hospital, Biratnagar, Morang Nepal; 22 Department of Community Medicine, Maharajgunj Medical Campus, Institute of Medicine, Tribhuvan University, Kathmandu, Nepal; 23 Department of Cardiology, Shahid Gangalal National Heart Centre, Kathmandu, Nepal; 24 Department of Cardiology, National Academy of Health Sciences, Bir Hospital, Mahaboudha, Kathmandu, Nepal; 25 School of Public Health, Central University of Nicaragua, Managua, Nicaragua; 26 School of Public Health and Community Medicine, B.P. Koirala Institute of Health Sciences, Dharan, Nepal; 27 Health Coordination Division, Ministry of Health and Population, Government of Nepal, Kathmandu, Nepal; 28 Teaching & Training Unit, Division of Infectious Diseases and Tropical Medicine, University Hospital, LMU, Munich, Germany; 29 Center for International Health (CIH), Ludwig-Maximilians-Universität, Munich, Germany; 30 Department of Pharmacy, Manmohan Memorial Institute of Health Sciences, Kathmandu, Nepal; 31 Department of Community Medicine, Kathmandu University School of Medical Sciences, Dhulikhel, Kavre Nepal; 32 Department of General Practice and Emergency Medicine, Kathmandu University School of Medical Sciences, Dhulikhel, Kavre Nepal; 33 Department of Pharmacology, Kathmandu University School of Medical Sciences, Dhulikhel, Nepal; 34 Pharmacovigilance unit/ Research and Development Division, Dhulikhel Hospital Kathmandu University Hospital, Dhulikhel, Kavre Nepal; 35 Policy, Planning and Monitoring Division, Ministry of Health and Population, Government of Nepal, Kathmandu, Nepal; 36 Department of Nursing, Kathmandu Medical College and Teaching Hospital, Kathmandu, Nepal; 37 School of Nursing, Faculty of Health, Queensland University of Technology, Brisbane, Queensland Australia; 38 Center for Methods in Implementation and Preventive Science and Department of Biostatistics, Yale School of Public Health, New Haven, USA; 39 Department of Public Health, Nepal Institute of Health Sciences, Stupa Health Care Center Cooperative Limited, Jorpati, Kathmandu, Nepal; 40 Department of Nursing, Nepalgunj Nursing Campus, Institute of Medicine, Tribhuvan University, Banke, Nepalgunj, Nepal; 41 Global Health Institute, Sun Yat-Sen University, Guangzhou, China; 42 Global Health Research Center, Duke Kunshan University, Kunshan, China; 43 Peking University School of Global Health and Development, Beijing, China; 44 Dean, Kathmandu University School of Medical Sciences, Dhulikhel, Kavre Nepal; 45 Department of Medicine (Cardiology), Dhulikhel Hospital Kathmandu University Hospital, Dhulikhel, Kavre Nepal

**Keywords:** Health system performance, CVDs, Nepal, Health system building blocks

## Abstract

**Background:**

Cardiovascular diseases (CVDs) are the leading cause of deaths and disability in Nepal. Health systems can improve CVD health outcomes even in resource-limited settings by directing efforts to meet critical system gaps. This study aimed to identify Nepal’s health systems gaps to prevent and manage CVDs.

**Methods:**

We formed a task force composed of the government and non-government representatives and assessed health system performance across six building blocks: governance, service delivery, human resources, medical products, information system, and financing in terms of equity, access, coverage, efficiency, quality, safety and sustainability. We reviewed 125 national health policies, plans, strategies, guidelines, reports and websites and conducted 52 key informant interviews. We grouped notes from desk review and transcripts’ codes into equity, access, coverage, efficiency, quality, safety and sustainability of the health system.

**Results:**

National health insurance covers less than 10% of the population; and more than 50% of the health spending is out of pocket. The efficiency of CVDs prevention and management programs in Nepal is affected by the shortage of human resources, weak monitoring and supervision, and inadequate engagement of stakeholders. There are policies and strategies in place to ensure quality of care, however their implementation and supervision is weak. The total budget on health has been increasing over the past five years. However, the funding on CVDs is negligible.

**Conclusion:**

Governments at the federal, provincial and local levels should prioritize CVDs care and partner with non-government organizations to improve preventive and curative CVDs services.

**Supplementary Information:**

The online version contains supplementary material available at 10.1186/s12913-021-06681-0.

## Introduction

Cardiovascular diseases (CVDs) are the leading cause of premature death and disease burden globally [1], disproportionately affecting low-and middle-income countries (LMICs) [2]. The South East Asian Region (SEAR) (including India, Pakistan, Bhutan, Bangladesh, Sri Lanka, and Nepal) is home to 20% of the world’s population and has one of the highest burdens of CVDs in the world [3]. The estimated economic loss due to CVDs in LMICs was 3.7 trillion US dollars in 2010, approximately 2% of the Gross Domestic Product of all LMICs [4].

Nepal is a lower middle-income country in South Asia with a per capita GDP of USD 1048 [5]. CVDs are the topmost cause of death and disability in Nepal, with an increase in both absolute and relative number of deaths over the past 16 years [6]. Ischemic heart disease was the primary cause of death accounting for 16% of total deaths in 2017 [7]. Premature deaths due to ischemic heart disease and stroke increased by 40 and 44%, respectively between 2007 and 2017 [8]. High systolic blood pressure accounts for 20% of early deaths and 15% of overall disease burden in adults aged 50–69 years old [1]. In 2019, approximately one fourth adult Nepalese had elevated blood pressure, 11% had elevated cholesterol, 20% were overweight or obese, 30% used tobacco, 98% did not consume sufficient fruits and vegetables and Nepalese consume 9.1 g of salt per day [9].

Nepal’s health system is more focused on maternal neonatal and child health and communicable diseases, and has failed to gather national-level attention on the alarming increase of CVDs. Promulgation of Nepal’s constitution of 2015 redesigned the health system with three tiers of autonomous government: a federal level, 7 provinces and 753 local governments [10]. The pre-constitution era’s health care network with district hospitals, primary health care centers and health posts has been taken over by the respective governments. However, CVDs care is not well integrated into the health system. A limited number of government and private tertiary level facilities, all of which are in major cities, provide care to CVDs patients. The Ministry of Health and Population (MoHP) has also endorsed a package of essential non-communicable diseases (PEN) focusing on cardiovascular disease risk estimates and management of hypertension and diabetes in 2016; however, the program has reached 50 out of 77 districts [11].

The health system must gear up to meet the escalating healthcare needs of the growing burden of CVDs by expanding preventive and promotive services and upgrading the coordination between federal, provincial and municipal levels of facilities to provide curative services. This paper aims to perform comprehensive health system assessment to identify the major gaps in the health system performance (equity, access, coverage, efficiency, quality, safety and sustainability) of adult CVDs related to health care within six health system building blocks (governance, financing, service delivery, information system, human resources and medical products). This will help determine priorities and lead to the formation of specific, feasible, context-specific national goals for the prevention and management of adult CVDs.

## Methods

All methods were carried out in accordance with relevant guidelines and regulations. We formed a task force co-chaired by the executive chairperson of the Nepal Health Research Council (NHRC) and principal investigator from the Kathmandu University School of Medical Sciences (KUSMS). The task force constituted members from the Ministry of Health and Population, NHRC, a patient representative, a family member, and the research team. The task force steered the study and provided overall guidance to achieve its objectives.

### Study design

This was a mixed method cross-sectional study to obtain insight into local practices and circumstances from policy makers and program planners’ interviews and documents, and to provide a comprehensive perspective on gaps between theory and practice of CVDs prevention and management. We investigated the health system performance using the United States Agency for International Development (USAID) manual “The Health System Assessment Approach: A How-To Manual”, Version 2.0 [12]. The health system assessment manual is a comprehensive tool to assess a health system’s ability to effectively undertake functions within six health system building blocks: leadership and governance, health financing, human resources for health, health service delivery, health information systems, and medical products and technologies.

### Data collection

We collected data in two phases. In the first phase, we reviewed official documents, both published and unpublished, which provided details of Nepal’s health system. We covered the six-health system building blocks, with a specific focus on non-communicable diseases (NCDs) in general, and CVDs in particular. We reviewed 125 documents including legal documents, policies and regulations, guidelines, reports, and websites. The complete list of documents is available in supplement A. In the second phase, we purposefully selected policymakers and implementers at the central level for key informant interviews to explore the perception of gaps in the health system to deliver CVD services in Nepal in terms of equity, access, efficiency, quality and sustainability. We interviewed 52 key informants including policy makers, university deans, professional council representatives, civil society members, cardiovascular health care providers, pharmacists, and a health economist. We interviewed the key informants in a private location in the Nepali language except 1 (participant preferred English) using a pre-tested interview guide. We received ethical approval from the Ethical Review Board (ERB) of the Nepal Health Research Council (#176/2018) and obtained written informed consent from the participants for interview and audio recording.

### Data analysis

We gathered information from all possible documentary sources, and analyzed and grouped all the information separately for each individual indicator of all six building blocks. We transcribed all the Key Informant Interview (KII) audio recordings into Nepali verbatim and an investigator compared audio against the transcripts. We inductively generated meaningful codes from the transcripts and used thematic analysis [13] to analyze the findings. Two investigators independently coded all the transcripts, the intercoder agreement was 89%. We categorized dominant and significant codes into thematic elements evaluating health system performance on access, equity, efficiency, quality, and sustainability across the six building blocks of the health system using a matrix as presented in Table 1.

**Table 1:** Health system building block and function matrix adapted from USAID manual of health system assessment

Building Block	Equity	Access	Efficiency	Quality (including safety)	Sustainability
Governance	Health policy ensuring equity	Information on quality of care is available to the population	Role of civil society including professional organizations to oversee health services and follow protocols, standards, and code of conduct	Regulations (protocols, standards, code of conduct, and certification) are known and enforced	Organized and financed in a way that offer incentive to public, NGOs and private providers
Financing	Government and out of pocket expenditure as % of total health expenditure Budget allocation for CVD Health insurance mechanism User fee policy	Per capita total expenditure in health Health insurance coverage Service covered by health insurance	Local-level spending authority and institutional capacity	Provider payment mechanism for health insurance	External resources as percent of total health expenditure Sustainability of health insurance Provider payment mechanism
Service Delivery		Availability of CVD services Time to reach nearest health facility	–	Existence of clinical standards into a practical form that can be used at local level	–
Human Resource	Ratio of health personnel per 1000 Ratio of healthcare workers distribution by cadre, geography and sector	–	Existence of costed HRH strategic plan; evidence that the plan is being implemented	Enabling environment exists for health professionals (job description, tools, supplies, supportive supervision)	Active stakeholder participation in human resource policy and processes
Medical products	Out of pocket expenditure on medicine	Percent of household within 30 min reach to public facilities that dispense essential medicine	Percent of procurement according to plan	System of collecting data regarding efficacy, quality, and safety of marketed products	System to recover pharmaceuticals dispensed in public facilities
Information system		–	Use of data for planning, budgeting or fund raising	Presence of procedure to verify the quality of data	Availability of financial and/or physical resources to support information system

### Stakeholder engagement

We engaged a wide range of stakeholders from different sectors of CVD care within the 7Ps framework [14] (Patients and the Public, Providers, Purchasers, Payers, Public Policy makers and Policy advocates, Product Makers, and the Principal Investigators) in various steps of the needs assessment. At our first stakeholders meeting, we explained our plan, discussed our protocol and received recommendations on improving research methods and design [15], which we implemented in our final protocol before data collection. After completing the first phase of data analysis, we conducted a second stakeholders meeting with the same representatives to disseminate and validate our information. The recommendations are incorporated in our final findings of needs assessment.

## Results

We reviewed 12 legal documents, 10 policies and regulations, 14 guidelines, 65 reports, and 21 websites (Supplement B). We interviewed 52 key informants including policy makers (35%), university deans (8%), professional council representatives (6%), civil society members (6%), cardiovascular health care providers (28%), pharmacists (15%), and a health economist (2%). The mean age was 45 ± 9 years, 37% of the participants were female and 87% had a Master’s degree or higher level of education. Half of the respondents had working experience of more than 20 years.

### Health system building blocks for CVD

The description of the health system building blocks for cardiovascular disease prevention and management is presented in Table 2.

**Table 2:** Health System building blocks on CVD prevention and care in Nepal

Building Blocks	CVD prevention and care
Governance	Nepal underwent a major political change in 2015 resulting in a restructuring of health-care systems with local empowerment. However, health care responses across the country vary substantially. The historical centralized governance of health potentially still survives in many parts of the country, whereas in some parts of the country, health care is governed by local governments. Private sector is the major provider of cardiovascular health-care. Nearly all private facilities offer CVD services compared to only 71% of public facilities [67], data specifically for CVD care were not available due to lack of CVD reporting in routine HMIS. Non-governmental organizations (NGOs) are a big part of the healthcare system, but have not yet played a major role in CVD care, except for Rheumatic Heart Disease.
Financing	The Government of Nepal spending on health care accounted for only 26.7% of the total health expenditure (THE) in FY 2015/16 [18]. The total spending on CVD was USD 2.8 million (2% of current health expenditure, 0.19% of THE) in the year 2015/16.
Service Delivery	National Health Policy 2017 ensures access to Universal Health Coverage to every citizen through a network of 836 hospitals including 2 public Cardiac specialized hospitals, 278 primary health centers, and 3840 Health posts [22]. National clinical guidelines on CVDs are not available [68]. Two major public cardiovascular disease hospitals (Sahid Gangalal National Heart Centre, and Manmohan Cardiothoracic Vascular and Transplant Center) are situated in the capital city. MoHP initiated PEN in February 2017 which covers screening, diagnosing, treating and referring services for CVD risk and hypertension at health posts, primary healthcare centers of 30 districts (out of 77 districts) of Nepal [69].
Human Resources	The ratio of doctors and nurses per 10,000 population is 8.9 and 20.8, respectively [20]. In 2019, 176 doctors were registered as cardiologist (154) or cardiothoracic surgeon (22) in Nepal (0.006 cardiologist/1000 population) [70].
Medical Products	The DDA regulates all functions relating to controlling the production, marketing, distribution, sale, export, import, storage and use of drugs [71]. There are 359 drugs listed on the National Essential Medicine List 2016. The government provides 70 of them for free as National Free Essential Medicines, which includes 9 CVDs drugs (Aspirin, Adrenaline, Atenolol, Amlodipine, Digoxin, Enalapril, Furosemide, Atorvastatin and Atropine) [72].
Information System	The Health Management Information System (HMIS) collects all health-related information from public and private health care. Only nine health conditions (Acute Rheumatic Fever, Bronchial Asthma, COPD, Cardiac failure, Heart failure, Hypertension, Ischemic heart disease, Rheumatic heart disease and other cardiac related conditions) are grouped under the cardiovascular and respiratory related problems. The national HMIS does not systematically collect and analyze CVD risk factors and CVD conditions and is not presented as a separate chapter in the annual health report [73].

### Health system performance and gap in CVDs prevention and care

The status of health system performance in terms of equity, access, efficiency, quality and sustainability are summarized below. The major health system gaps are presented in Table 3.

**Table 3:** Health system gaps to deliver CVD prevention and management service

	Major Gaps
Equity	● More than 50% of the total health expenses are covered out-of-pocket, indicating a huge financial burden to the general population. ●The government budget for NCD including CVD is only 0.2% of the total government budget in health even though CVD is the topmost cause of death and disability in Nepal [6]. ● Significant HRH gaps across all health cadres, and with nurses in particular. Doctor and nurse distribution is skewed toward private sector and urban centers. ● CVD services such as surgery and INR are available in the central tertiary hospitals.
Access	● Only 10% of the population is covered by national health insurance. ● Only 30 (out of 77) districts have PEN program that provide primary CVD care services. ● Specialty CVD care services are concentrated in urban areas ● One-fourth of the households in the mountains and 17% in hills are 60 min or more away from the nearest health facility.
Efficiency	● Shortage of human resources for CVD care. ● Procurements have lengthy administrative processes.
Quality (including safety)	● No national guidelines on clinical management of CVD ● Inadequate infrastructure and equipment for CVD care ● Medical products quality is not well monitored despite having policies and guidelines in place because implementation challenges such as limited training on guidelines, lack of supervision and monitoring, and resources constraints.
Sustainability	● Although there is policy to provide grants based on the performance, the policy is not functioning well. ● External resources covered 11% of the health spending. ● Health insurance premium collection is not sufficient as only 10% of the population are insured.

#### Equity

##### Health policy:

It is a constitutional right to have free basic health care from the state [16]. Basic health services for CVDs includes risk identification, initial examination and management, lifestyle counselling and referral for pre-hypertension, uncomplicated hypertension, impaired blood glucose and uncomplicated diabetes [17]. NCD intervention policies related to Behavior change communication, which are the primary/primordial prevention, are more prioritized with regards to food reformulation, taxation, mostly due to resistance and economic power of the corporate community.

##### Health expenditure:

General government expenditure on health was 27% of the total health expenditure in 2016 [18]. In the same year the out-of-pocket (OOP) expenditure for healthcare was 52% of the total health expenditure - 63% of this expenditure was incurred for medicines and medical goods. Of the 60% total OOP spending on the diseases/health categories, almost half was spent on NCDs [18].

##### Budget allocation for CVDs:

The government direct budget on the NCD programs was USD 1.3 million (0.2% of the total government budget on health) in 2016 [19]. Twenty-nine percent of the budget was allocated to the central level and 69% was allocated to provincial level for procuring equipment, developing guidelines and manuals, and training health workers on PEN. Only 2% of the budget went to the municipality level for the raising awareness of NCDs in the community. Although this was a small proportion of the health budget, a representative from the NCD section at Epidemiology and Disease Control Division (EDCD) regarded this as a positive sign.

“*the NCD budget in comparison to the disease burden is very low.... But, it is still satisfactory, because in the past (three years ago) there was none*.” - NCD Section, EDCD, DoHS, MoHP representative

##### Health professional distribution:

The ratio of doctors and nurses per 10,000 population is 8.9 and 20.8, respectively [20]. The distribution of health professionals by provinces is presented in Table 4. Sixty percent of the doctors, 80% pharmacists and 38% of nurses are working in the private sector [21].

**Table 4:** Registered Health professionals’ density distribution per 10,000 population based on provinces [51]

Provinces	Provincial Population	Doctor Population Ratio	Nurse Population Ratio	Ayurveda HWs Population Ratio	Pharmacist Population Ratio	Health Lab Professional Population Ratio
Province 1	4,534,943	4.55	22.18	1.02	3.46	8.17
Province 2	5,404,145	5.83	6.82	2.66	2.34	6.22
Province 3	5,529,452	15.77	34.43	0.79	5.56	8.09
Gandaki	2,403,757	8.49	37.43	1.9	7.15	10.78
Province 5	4,499,272	4.68	19.29	1.62	3.52	7.9
Karnali	1,570,418	1.28	7.73	1.03	1.72	9.34
Sudurpaschim	2,552,517	1.72	6.26	1.39	2.48	6.80

#### Access

##### Availability of CVDs service:

Regular health services are delivered across the country through a network of 429 government hospitals (including 2 specialized cardiac centers), 407 private hospitals, 321 polyclinics, 409 registered clinics, 278 primary health care center, 496 urban health centers, 3840 health posts, and 289 rural community health units in Nepal [22]. Primary CVD care through the package of non-communicable diseases that includes CVD risk assessment and management, hypertension and diabetes screening and treatment are available in health facilities in 39% of the districts [23]. CVDs diagnosis and treatment services are available in tertiary level hospitals, concentrated in urban areas [24]. Referral pathways are not clearly defined - CVDs patients from remote areas get delayed services incurring high prices for travel.

*“Many of the heart attack patients from remote areas arrive too late to our healthcare system.” -*A tertiary hospital representative.

*“People who come from remote areas have to travel for days spending about USD 90 (on indirect medical cost) to get to our hospital (a specialized cardiac center located in Kathmandu) just for a blood test called International Normalized Ratio (INR), which costs USD 1.3 …...It doesn’t make sense.” -* National Heart Centre representative.

##### Time to reach a health facility:

Almost half of the population must travel more than 30 min to reach the nearest health facility, and 11% are at least 60 min away (Table 5). To address this, public primary health facilities conduct 3–5 outreach clinics per month within 30 min walking distance for their catchment populations. Eleven percent of the total health service users were covered by outreach clinics in 2017/18 [23]. However, CVD screening or care services are not usually offered by the outreach clinics.

**Table 5:** Percentage of households with nearest health facility

Geography	< 30 min	30–60 min	60+ minutes	Don’t Know
Mountain	34.5	39.9	25.3	0.2
Hill	39.4	42.1	17.4	1.1
Terai	61.5	35.3	3.1	0.0
Total	49.3	38.8	11.3	0.5

##### Health insurance:

National health insurance covers 36 CVD-related services (Supplement B) including inpatient, outpatient and emergency consultations, laboratory tests of blood, urine, ECG and echocardiography [25]. However, only 10% of the total population is covered by health insurance [26]. Hypertension was the third most common morbidity for health insurance use in 2019 [26].

##### Government subsidy:

The Nepali government provides free valve repair and replacement surgery post-Rheumatic Heart Disease to the citizens under 14 years of age and supports senior citizens (65 years and older) for heart surgery for up to USD 837 [27]. This amount covers only a part of the total expense though. For example, a valve replacement and repair would cost about USD 2500. And, these government programs are not well communicated with the public either.

*“I think that …. 1-2% of the population know about these schemes, most people (who come here for treatment) do not have any idea about government schemes”* - A tertiary hospital representative.

The government provides a subsidy of about USD 945 per person for health care for the ultra-poor. Ultra-poor are identified through a recommendation by the municipal authority. However, there is no system to monitor the effective and appropriate use of the subsidy.

“*Anyone can claim for Bipanna Nagarik (ultra-poor) if they bring the recommendations. There is no clear definition of bipanna nagarik. There should be one.” -*DoHS, MoHP representative.

#### Efficiency

##### Role of professional councils, civil society:

Professional councils regulate health care workers’ license and adherence to code of conduct, standards and protocols. Patients and the public can report medical malpractice, unfair pricing patterns, discrimination and abuse to professional councils. The council investigates the complaints and if found guilty may cancel the license, revoke certificates or prohibit the medical practice [19, 21]. ^.^

Three active civil societies (Cardiac Society of Nepal, Nepal Heart Foundation, Jayanti Memorial Trust) take initiatives and make continuous effort to interact with the government on planning and implementation of CVDs.

“*They (the government) have not asked us for any suggestions, we have to go to them and get involved. …..the NCD (NCD section, department of health services), we have never been once invited there for any discussion” -* Civil Society representative.

##### Local level spending authority and capacity:

A local health facility operation management committee (HFOMC) manages the revenue generated by the facilities, including revenue collected from user fees [28]. The capacity of the HFOMC, however, varies across the country.

*“Some HFOMC perform really well and are self-sustained, but some are completely lost. It depends a lot on local political environment” -* MoHP representative.

##### Human resource for health (HRH) strategic plan:

Human resources for health plan [29] has not been updated despite a federal decentralization of health structure in 2015. Nepal has a substantial shortage of healthcare workers for CVD care. Training non-physician health care workers can be an effective strategy to address the shortage and extend CVDs care into rural areas.

*“Task shifting will be really practical and feasible, the quality of care will increase. This is a very necessary step to be implemented, we must do it, must. Given the number and distribution of cardiologists in Nepal, it is clear that cardiologists alone cannot handle all the patients” -* Civil Society representative.

##### Procurement of medical products:

The logistic management section at the DoHS prepares an annual consolidated procurement plan and process within a 90–120 days lead time. Eighty percent of the planned procurement was completed in 2018 [30]. Out of 70 free essential drugs (9 are CVD drugs), the logistics management section procured 34 essential drugs (including 2 CVD drugs) in the year 2018 [31]. The procurement process was considered to be administratively lengthy.

*“Public procurement acts and rules are time-consuming and difficult to process ….” -* Logistic Management Section, DoHS representative.

##### Use of data for planning, budgeting and generating funds:

The health management information section at the DoHS uses an “information cycle” consisting of six steps - data collection, processing, analysis, presentation, interpretation and use. The data are compiled, managed and analyzed and presented to the planners and stakeholders quarterly at district-level and annually at provincial and federal-level [32]. However, the use of data is limited.

“*We train people, we generate information and forward it to them (planners), ..... but they hardly use these data. ...they (program implementers) use it only at the end of the year. ..They don’t use the data regularly to monitor the program” - DoHS representative.*

##### Provider payment mechanism for health insurance:

All health care services are cashless for beneficiaries with no co-payments [33]. The health insurance board is required to pay health facilities for the services they provide within 15 days of a claim. In practice, the payments are not made on time.

*“We have to wait for months to get reimbursed for the insurance. I have heard stories where institutions were not reimbursed for more than a year.” - Tertiary care hospital representative.*

#### Quality (including safety)

##### Guidelines on CVDs:

There are no national clinical guidelines on treatment of CVDs. The PEN protocol is used for the prevention and management of NCD including CVDs in primary health centers in Nepal through health assistants and nurses. The PEN program has covered only 30 districts. However, due to decentralization of the health system after 2016, the trained health workers have been transferred to other districts, which has limited the PEN implementation, supervision and monitoring. The NCD section at the department of health services plan to scale up the PEN to the rest of the country. The implementation of the PEN package has not been evaluated.

*“We trained all health workers on PEN, but we don’t know how many are still working in their station and how many are actually following the guidelines. They are not reporting for sure, as we do not receive the data.” -* NCD section representative.

##### Enabling environment:

MoHP has defined several acts and policies to provide an enabling environment for health workers at healthcare institutions and to ensure strategic planning, deployment and development of healthcare workers [34]. The annual staff performance evaluation and operation management survey evaluates the performances of all staff and institutes in governmental organizations. However, in practice, the majority of these acts, policies and procedures are not functioning effectively. Performance appraisal is not standard and rewards are not performance-based. In addition, inadequate infrastructure and inadequate equipment demotivate the human resources in public sectors.

*“Can a doctor work alone? She/he needs an ECG machine, X-ray machine, lab for TC-DC (total count-direct count), accommodation. internet to keep updated. Concerned bodies must arrange these.” -* An academic institution representative.

*“The job is secured and there is no performance-based evaluation that is affecting their quality of work... If staff gets paid and promoted even when they do not work, then who would want to work hard?” -* Policy, Planning and Monitoring division representative, MOHP.

*“There is no transparency in the evaluation. If you can flatter your senior, you get higher marks in your evaluation and get promoted. The problem is not in our tools but the way they are implemented in the system” -*MoHP representative.

##### System to assess quality of medical products:

The Department of Drug Administration (DDA), along with the National Medicines Laboratory (NML) conducts post-marketing surveillance (PMS) to assess the quality and safety of the drugs [35]. PMS, however, is not very extensive due to the lack of infrastructural and human resources.

##### Verify the quality of data:

The DoHS conducts a routine data quality assessment with an inbuilt feedback mechanism using District Health Information System 2.3 software [36]. However, the implementation of data collection and analysis is poor.

*“The problem is: there are no technical staff dedicated to do this work (data verification).”* - Health management and information system section, DoHS representative.

#### Sustainability

##### Incentive to public, NGOs and private providers:

There is a policy to provide grants to the public, NGOs, and private health facilities based on their performance [37]. However, the policy has not been implemented. The number of facilities evaluated and those who got grants are not systematically documented.

##### Financing:

The health sector budget has increased over the last six years from USD 321 million in 2016 to USD 759 million in 2020 [38]. External resources for health were 11% of the total health spending in the year 2016 [18]. The government has provided a grant of USD 173,000 to cover health insurance expenses [33].

*“At this initial phase (National Health insurance started in 2015), the government’s grant is sufficient for the health insurance to cover the cost and we don’t have to use premium collected. But, going forward, premium collected will determine our sustainability.” -* Health Insurance Board representative.

##### Resources for information system:

In 2019, 0.36% of the total health budget was allocated for the information system [23, 39]. The transitioning of paper to electronic based data recording and reporting systems is a priority. However, this has been hindered by inadequate physical facilities such as computers, electricity, and internet [23, 35].

## Discussion

This comprehensive health system assessment has revealed that the health system is poorly prepared to address the large and growing CVD burden in Nepal. Primary CVDs health care in the form of PEN package care is available in 37% of the country. Specialty cardiac services are concentrated in urban areas. National health insurance, that covers 36 CVDs care services, offers a promising step for equitable CVDs prevention and management services. However, only 10% of the population is enrolled in the insurance program. The efficiency of CVD prevention and management programs in Nepal are affected by a substantial shortage of human resources, weak monitoring and supervision, and inadequate engagement of stakeholders. There are policies and strategies in place to ensure the quality of care, however, their implementation and supervision is weak. The total budget for the health sector has been increasing over the past five years. However, the funding on CVDs is negligible.

Although Nepal has a network of health facilities from the primary to tertiary level, access to CVDs prevention and care is limited. The recent government initiative to implement the PEN package in the primary health system is promising. The nationwide scale-up requires a well-integrated training of health workers, strengthening service delivery and establishing a monitoring and evaluation mechanism [40]. A critical element of ensuring access to CVD care is to be cognizant of the diseases of poverty, notably rheumatic heart disease [41]. Nepal stands out compared to other countries for its unique provisions of free valve repair and replacement surgeries for rheumatic heart disease (RHD). However, access by the most vulnerable groups is still limited because these services are only available in tertiary hospitals in the capital city and rural populations are not aware of these programs. The health service readiness to provide cardiovascular diseases is suboptimal in public facilities [42].

Health care equity can be advanced by the programs that incorporate universal population-level strategies with targeted approaches for at-risk groups [43]. The national health insurance that covers 36 CVDs services offers a promising step for equitable CVDs prevention and management services, but covers only a tenth of the population. It is important to note that only 27% of the total healthcare expenditure is paid by the Government while 52% is out-of-pocket. High out-of-pocket healthcare expenditures threaten people with low economic status due to impoverishment. Catastrophic healthcare expenses for secondary CVD treatment and the resulting impoverishing effect have already been documented in many low- and middle-income countries including Nepal [44]. The government supports the ultra-poor with USD 945 per surgery, but it is not enough. The cost of surgery can be 4.5 times higher [45]. The costs of providing primary CVD management care in Nepal is estimated to be USD 1.86 per capita, approximately 0.2% of the per capita gross national product [46]. The acute shortage of healthcare providers limits the capacity of LMICs to manage CVD at the primary care level [47].

In Nepal, there are 8.9 physicians and 20.8 nurses per 10,000 population. This is low compared to the WHO recommendation of 10 physicians and 40 nurses per 10,000 population [48] [49]. The respondents suggested task shifting as a strategy to address the shortage as well as the inequitable distribution of the health workforce. Task shifting is a potentially viable and low-cost strategy for reducing the growing CVD epidemic in LMICs because it utilizes multiple strategies that are amenable to the management of CVDs including screening, counselling on lifestyle modification, initiation of treatment and referral to specialist care [50]. In Nepal, task-shifting might also be more feasible due to the high density of paramedics in the population (27.6 paramedics per 10,000) [51].

Our findings highlighted the existence of limited national guidelines and clinical protocols for CVDs treatment. Absence of clinical guidelines are reasons for insufficient implementation of evidence-based CVD prevention and treatment [52]. Practitioners and patients are likely to adopt and adhere to protocol-directed management and therapy, which encompasses clinical evaluation, diagnostic testing, and both pharmacological and procedural treatments [53]. In Nepal, the intact policies and procedures provide ample opportunity to control the quality of drugs and medical products. However, the implementation of these policies is challenging. An estimated 30% of the world’s medicine regulatory authorities have poor capacity to monitor drug quality [54]. This limitation likely has a significant adverse impact on population health. Wide range of price variation exists and counterfeit medicines, possibly for CVD, are available rampant in the market [55]. In 2010, 20 million fake and illegal medicines were seized in South East Asia [56].

It is encouraging that over the past six years, the health sector budget increased 2.3 times in Nepal. External development partners supported 11% of the total health spending. However, CVD is not a priority health program. The budget for NCD programs was only 0.2% of the government health budget. Sustainability may be strengthened by expanding the health insurance program. It would be necessary to integrate CVD care into already existing primary care systems to more effectively deliver the care including screening and treatment, improved health record, cost-effective drug distribution, prohibitive health care costs, and basic health surveillance [57].

This study provides an in-depth analysis of adult CVD prevention and management gaps in Nepal. To our knowledge, this is one of the first comprehensive health systems gap analysis for CVDs in Nepal. The assessment was conducted with a participatory and transparent approach which allowed key stakeholders at government, CVDs, patient representatives, non-government and civil societies to contribute to the assessment process. The recommendations drawn from the assessment are aimed at providing guidance to national and international partners on strengthening the health system. The study had several limitations. First, we reviewed the documents that were available publicly or could be accessed through professional connections. Although we believe that the list of documents was comprehensive, it is possible that we have missed some. Second, we used secondary data and we were not able to verify them due to resource constraints. Third, there may have been some social-desirability bias as we interviewed focal persons of respective sections of the DoHS, MoHP. Fourth, the data on CVD services availability and use were not available. Fifth, assessing access was limited because of the lack of data on availability and geographical distribution of CVD services such as ECG, echocardiography, Cath Labs, and human resource (cardiologists and cardiac surgeons). Finally, we could not extend data collection to the provincial or municipal level government and people living with NCDs.

### Recommendations

We recommend the Government of Nepal to urgently prepare a national health system strategy to address CVD prevention and treatment to:

1. Increase access: Government should increase spending on primary CVD prevention, screening and management strategies to reduce the disease burden, as well as to protect the poor from economic hardship and financial shock [58]. With the nation-wide health system network, there are opportunities to focus on programs and policies that are cost-effective. Ideally, this would involve a bimodal approach in which evidence-based clinical strategies for CVDs prevention and treatment are complemented by evidence-based population level strategies [57, 59]. Government should evaluate and expand the PEN package that enables health workers to target those who are at the highest risk for heart attacks and strokes [60, 61].
2. Ensure Equity: Government should rapidly expand insurance programs and availability of CVDs services for ensuring equity in CVDs prevention and management. Collaborations among providers, policymakers, researchers, and other stakeholders can facilitate this process by addressing implementation challenges, monitoring the policy impacts on vulnerable populations, and advocating for funding [43].
3. Improve efficiency: Government should build human resource capacity for CVDs in partnership with non-state providers and academic institutions. CVD care can be efficiently and rapidly expanded by shifting preventive care tasks to non-physician and non-nurse health workers such as paramedics and community pharmacists with clearly defined roles, evaluation, on-going training, and supervision [62]. In addition, integrated electronic decision support systems can increase cardiovascular risk assessment and data use [63, 64].
4. Control quality: Government should create a multidisciplinary task force to standardize treatment and care of CVDs through national level protocols, guidelines and regulations. In addition, risk-based post-marketing quality surveillance programs, supporting tools, and communication strategies should be implemented [65, 66].
5. Strengthen sustainability: Government should strengthen health insurance to increase the coverage, expand the scope of CVD care and improve reimbursement process.

## Conclusion

Although CVDs cause the most deaths and disabilities among adults, Nepal struggles to address the gaps for CVD prevention and management. CVDs services are adversely affected by negligible government spending, low health insurance coverage, shortage of human resources, limited participation of stakeholders, and poor implementation of policies and strategies. Governments at federal, provincial and local level should prioritize CVD care and partner with non-government organizations, private sector, academia, and CBOs, to improve preventive and curative CVD services. Periodic health system assessment can help identify the gaps and build evidence-based strategy to ensure improved cardiovascular health.

## Supplementary Information

**Additional file 1.**

## Data Availability

The data is available on reasonable request. Source/ contact person: Archana Shrestha - archana@kusms.edu.np

## Abbreviations

CVD: Cardiovascular Diseases
DoHS: Department of Health Services
ECG: Electrocardiogram
GDP: Gross Domestic Product
HFOMC: Health Facility Operation Management Committee
HMIS: Health Management Information System
HRH: Human Resource for Health
KII: Key Informant Interview
LMICs: Low-and Middle-Income countries
MoHP: Ministry of Health and Population
NCDs: Non-Communicable diseases
NGO: Non-Governmental Organization
NML: National Medicines Laboratory
PEN: Package of Essential Non-Communicable Diseases
RHD: Rheumatic Heart Disease
USD: United States Dollar
