# Supplementary material for: Health system gaps in cardiovascular disease prevention and management in Nepal
Source: BMC Health Serv Res. 2021 Jul 5;21:655. doi: 10.1186/s12913-021-06681-0 (PMC8258928; doi:10.1186/s12913-021-06681-0)
Supplement: Supplementary file 1 — Additional file 1. [file 12913_2021_6681_MOESM1_ESM.docx]

**Supplement A: List of documents reviewed**

| **Legal Documents: Acts, Rules and Laws** | |
| --- | --- |
| **S.N.** | **Particulars** |
| 1. | Department of Drug Administration. Drugs Act 2035. http://www.dda.gov.np/content/drugs-act-2035. Published July 2017. |
| 2. | Public Procurement Monitoring Office. The Public Procurement Act, 2007. https://ppmo.gov.np/index.php?_route_=acts_and_regulations. Published January 2007 |
| 3. | Nepal Law Commission. Security of the Health Workers and Health Organizations Act, 2066 (2010). http://www.lawcommission.gov.np/en/wp-content/uploads/2019/07/The-Public-Health-Service-Act-2075-2018.pdf. Published May 2009 |
| 4. | Nepal Law Commission. The Public Health Service Act, 2075 (2018). http://www.lawcommission.gov.np/en/wp-content/uploads/2019/07/The-Public-Health-Service-Act-2075-2018.pdf. Published September 2018 |
| 5. | Nepal Law Commission. Security of the Health Workers and Health Organizations Act, 2066 (2010). http://nepalpolicynet.com/images/documents/publichealth/acts/security-of-the-health-workers-and-health-organizations-act-2066-2009-e.pdf. Published May 2009 |
| 6. | Nepal Law Commission. Public Service Commission Act 2066, (2010). http://nepalpolicynet.com/images/documents/publichealth/acts/security-of-the-health-workers-and-health-organizations-act-2066-2009-e.pdf. Published January 2010 |
| 7. | Nepal Government Health Insurance Board. Health Insurance Act 2074. https://hib.gov.np/np/detail/b-ai. Published October 2017 |
| 8. | Nepal Law Commission. Nepal Medical Council Act 2020, (1964). http://www.lawcommission.gov.np/en/archives/category/documents/prevailing-law/statutes-acts/nepal-medical-council-act-2020-1964. |
| 9. | Nepal Law Commission. Nepal Nursing Council Act, 2052 (1996). http://www.lawcommission.gov.np/en/archives/category/documents/prevailing-law/statutes-acts/nepal-nursing-council-act-2052-1996. Published March 1996 |
| 10. | Nepal Law Commission. Nepal Health Professional Council Act, 2053. http://www.lawcommission.gov.np/en/archives/category/documents/prevailing-law/statutes-acts/nepal-health-professional-council-act-2053. Published February 1997 |
| 11. | Nepal Law Commission. Nepal Pharmacy Council Act 2000. http://www.lawcommission.gov.np/en/wp-content/uploads/2018/10/nepal-pharmacy-council-act-2057-2000.pdf. Published January 2001 |
| 12. | Constitution of Nepal.; 2015. http://www.lawcommission.gov.np/en/archives/category/documents/prevailing-law/constitution/constitution-of-nepal |
| 13. | Nepal Law Commission. Drugs Registration Rules, 2038 (1981). http://www.dda.gov.np/content/drug-registration-regulation-2038. Published August 1981. Updated September 2001 |
| 14. | Nepal Law Commission. Drug Investigation and Inspection Rules, 2040 (1983). http://www.dda.gov.np/content/drug-investigation-and-inspection-rules-2040. Published August 1983. Updated January 1991 |
| 15. | Nepal Law Commission. Drug Category Rules, 2043 (1986). http://www.dda.gov.np/content/drug-standard-regulation-2043. Published August 1986 |
| 16. | Nepal Law Commission. Nepal Medical Council Rules, 2024 (1968). https://nmc.org.np/nmc-regulation-2024-1968#. Published January 1968 |
| 17. | Nepal Law Commission. Constitution of Nepal – Part 3, Article 35.; 2015. Accessed July 31, 2020. http://www.lawcommission.gov.np/en/archives/category/documents/prevailing-law/constitution/constitution-of-nepal |
|  |  |
| **Policies and Regulations:** | |
| 1. | Ministry of Health and Population. Nepal’s Medicines Policy- 2007. https://www.medbox.org/document/nepal-national-medicines-policy-2007#GO. Published 2007 |
| 2. | Ministry of Health and Population. National Health Policy 2071. http://www.dda.gov.np/content/national-health-policy-2071. Published 2014 |
| 3. | International Cancer Control Partnership. Integrated Non-Communicable Diseases (NCDs) Prevention and Control Policy of Nepal. https://www.iccp-portal.org/system/files/plans/integrated_non-communicable_diseases__ncds__prevention_and_control_policy_of__nepal.pdf. Published 2007 |
| 4. | Ministry of Health and Population. National Health Policy 2074. https://dohs.gov.np/wp-content/uploads/2014/04/NHP-2074_policy-01.pdf. Published October 2017 |
| 5. | Office of the Prime Minister and Council of Ministers. Health Insurance Board Regulations 2075. https://hib.gov.np/public/uploads/shares/nimabali.pdf. Published March 2019 |
| 6. | Nepal Law Commission. The Public Procurement Regulation, 2007. https://ppmo.gov.np/image/data/files/acts_and_regulations/public_procurement_rules_2064.pdf. Published August 2007 |
|  |  |
| **Guidelines:** | |
| 1. | World Health Organization. Guide to good storage practices for pharmaceuticals. https://www.who.int/medicines/areas/quality_safety/quality_assurance/GuideGoodStoragePracticesTRS908Annex9.pdf?ua=1#:~:text=Materials%20and%20pharmaceutical%20products%20should,from%20accumulated%20waste%20and%20vermin. Published 2003 |
| 2. | Department of Drug Administration. Guidelines for Drug Donation to Nepal. http://www.dda.gov.np/content/guidelines-for-drug-donation-to-nepal. Published June 2018 |
| 3. | Nepal Health Training Center. Health Facility Operation and Management Committee- A reference guideline for local level, 2063. https://drive.google.com/file/d/0BxYPsAJu5Bn_T01tLVFVNEdkRDQ/view. Published 2006 |
| 4. | Ministry of Health and Population. Bipanna Nagarik Awadhi Upachar Kosh Nirdeshika, 2075. https://drive.google.com/file/d/1N9tAiiDBkuxSqc0lZX8QN_l9F28LMjH0/view. Published October 2018 |
| 5. | Department of Drug Administration, Ministry of Health and Population. Medicine Registration Guidance. http://www.dda.gov.np/content/drug-registration-guidance-2073. Published 2016 |
| 6. | Health Management Information Section, Ministry of Health and Population. HMIS indicators 2070. https://dohs.gov.np/wp-content/uploads/2019/03/IHIMS-Indicators-booklet.pdf. Published March 2014 |
| 7. | Management Division, Ministry of Health and Population. Health Management Information System (HMIS) Recording and Reporting Forms 2070. http://nnfsp.gov.np/PublicationFiles/058fd41d-ccfa-4908-b876-6fc73dd05f6b.pdf. Published March 2014 |
| 8. | Management Division, Ministry of Health and Population. HMIS Data Analysis Use Book 2014. https://dohs.gov.np/hmis_data_analysis_use_book/. Published June 2014 |
| 9. | Management Division, Ministry of Health and Population. DHIS2 Software Operational Guideline Nepal. https://www.thecompassforsbc.org/sites/default/files/strengthening_tools/DHIS%20Mannual%20Nepal_Fianl.pdf. Published 2016 |
| 10. | Ministry of Health and Population. Routine Data Quality Assessment Guidelines, 2075. https://rdqa.mohp.gov.np/resources. Published June 2018 |
| 11. | Ministry of Health and Population. Guidelines for Health Institute Establishment Upgraded version, 2070. https://www.mohp.gov.np/downloads/Guideline%20for%20Health%20Institutions%20Established%20Upgrade%20standard.pdf. Published January 2014 |
| 12. | Ministry of Health and Population. Local Level Program Implementation Guidelines. https://www.mohp.gov.np/eng/news/265-local-level-program-guidelines. Published September 2018 |
| 13. | Ministry of Health and Population. TOR for Health Workers at Local Level. https://www.publichealthupdate.com/tor-of-health-workers-at-local-level/. Published 2016 |
| 14. | National Health Training Center. Health Facility Operation and Management Committee- A Reference Guideline for Local Level.; 2018. Accessed July 29, 2020. <http://www.nhtc.gov.np/images/1_HFOMC_Guideline_2075.pdf> |
| **Reports:** | |
| 1. | Ministry of Health and Population. Central Bidding and Local Purchasing: A Discussion Paper. https://drive.google.com/file/d/1XrnoNeFSIDdg79jb8BuC91OQJ25DjUFs/view?usp=sharing. Published May 2009 |
| 2. | Ministry of Health and Population. Nepal National Health Accounts 2012/13 to 2015/16. https://www.who.int/docs/default-source/nepal-documents/nepal-nha-2012-13-to-2015-16-ministry-of-health-and-population-june-2018.pdf?sfvrsn=64645c52_2. Published June 2018 |
| 3. | Ministry of Finance. Budget Speech of Fiscal Year 2018/19. https://mof.gov.np/uploads/document/file/speech_english_20180715091610.pdf. Published May 2018 |
| 4. | Department of Health Services, Ministry of Health and Population. Annual Report, Department of Health Services, 2073/74 (2016/17). https://dohs.gov.np/wp-content/uploads/2018/04/Annual_Report_2073-74.pdf. Published April 2018 |
| 5. | Department of Drug Administration, Ministry of Health and Population. National List of Essential Medicine Nepal (Fifth Revision) 2016. http://www.dda.gov.np/content/essential-drug-list. Published 2016 |
| 6. | World Health Organization. Nepal Pharmaceutical Profile 2017. https://apps.who.int/iris/handle/10665/274871. Published 2018 |
| 7. | Ministry of Health and Population. Revised List of Free Essential Medicines to be purchased for fiscal year 2075/76. https://dohs.gov.np/wp-content/uploads/2018/08/Free_Drugs_List.pdf. Published August 2018 |
| 8. | Logistics Management Division, Ministry of Health and Population. Logistics Management Division Three Year’s Report (FY 2071/72- 2073/74). http://dohslmd.gov.np/web/uploads/source/Logistics%20Report%20Inner.pdf. Published June 2018 |
| 9. | Logistics Management Division, Ministry of Health and Population. Quality Assurance, version 2.0. http://dohslmd.gov.np/web/uploads/source/chapter9.pdf. Published February 2015 |
| 10. | Logistics Management Division, Ministry of Health and Population. Procurement Handbook 2074. http://www.nhssp.org.np/Resources/PPFM/Procurement_Handbook2074.pdf. Published 2018 |
| 11. | Ministry of Health, New ERA, Nepal Health Sector Support Program (NHSSP), ICF. Nepal Health Facility Survey 2015. https://dhsprogram.com/pubs/pdf/SPA24/SPA24.pdf. Published January 2017 |
| 12. | Ministry of Health, New ERA, ICF. Nepal Demographic Health Survey, 2016. https://dhsprogram.com/pubs/pdf/FR336/FR336.pdf. Published November 2017 |
| 13. | Department of Drug Administration, Ministry of Health and Population. Nepalese National Formulary 2nd Edition. http://www.dda.gov.np/content/nepalese-national-formulary-nnf. Published 2010 |
| 14. | Ministry of Health and Population, World Health Organization. Nepal Pharmaceutical Country Profile. https://un.info.np/Net/NeoDocs/View/4794. Published September 2011 |
| 15. | Development Resource Centre. Evaluation on: Essential Drug Procurement and Distribution Program Under Free Health Services. https://www.npc.gov.np/images/category/essential_drug.pdf. Published July 2012 |
| 16. | Logistics Management Division, Ministry of Health and Population. Consolidated Annual Procurement Plan (CAPP) Fiscal Year 2017/18. https://www.nhssp.org.np/Resources/PPFM/Consolidated_Procurement_Plan_Aug2017.pdf. Published August 2017 |
| 17. | Caffrey M, Chilvers R, Martineau T. Human Resources for Health: Nepal Country Profile. Ministry of Health and Population (MoHP)/ World Health Organisation (WHO)/ Nepal Health Sector Support Programme (NHSSP); 2013. <http://www.nhssp.org.np/NHSSP_Archives/human_resources/HRH_Nepal_profile_august2013.pdf> |
| 18. | Ministry of Health and Population. Nepal Health Sector Strategy 2015-2020. https://nepal.unfpa.org/sites/default/files/pub-pdf/NHSS-English-Book-final-4-21-2016.pdf. Published April 2018 |
| 19. | Ministry of Health and Population, NHSSP. Human Resources for Health Strategic Plan 2011-2015. https://www.who.int/workforcealliance/countries/Nepal_HRHStrategicPlan_finaldraft.pdf. Published January 2012 |
| 20. | FMoHP and NHSSP (2018). Budget Analysis of Ministry of Health and Population FY 2018/19. https://www.nhssp.org.np/Resources/PPFM/Budget_Analysis_of_Nepal_Federal_MoHP_FY2018_19_Sep2018.pdf. Published September 2018 |
| 21. | Ministry of Health and Population. Job description of employees 1997. |
| 22. | Department of Health Services. Social health security division report 2075/76. https://hib.gov.np/public/uploads/shares/notice_hib/health_insurance_report_2075-76.pdf |
| 23. | MInistry of Health and Population. Overview of Public-Private Mix in Health Care Service Delivery in Nepal. https://www.yumpu.com/en/document/read/37499727/overview-of-public-private-mix-in-health-care-rti-international. Published June 2010 |
| 24. | Ministry of Health. Nepal National Health Accounts 2009/10 - 2011/12. https://www.mohp.gov.np/downloads/Nepal%20NHA%20Report_2009%20to%202012_Ministry%20of%20Health.pdf. Published December 2016 |
| 25. | Health Insurance Board. Payment Mechanism and Rates. https://hib.gov.np/public/uploads/shares/pages/anusuchi_9_(payment_mechanism_and_rates)upload.pdf. Published 2016 |
| 26. | Social Health Security Division. Annual Report of SHSP 2016/17. https://hib.gov.np/public/uploads/shares/downloads/Annual_Report_of_SHSP_2073_74_Nepali.pdf. Published September 2017 |
| 27. | Ministry of Health and Population. Progress Report on Partnership, Alignment and Harmonisation in the Health Sector 2013/14. http://www.nhssp.org.np/NHSSP_Archives/jar/2015/06Partnership_JAR_report_february2015.pdf. Published February 2015 |
| 28. | Ministry of Finance. Budget Speech of Fiscal Year 2014/15. https://mof.gov.np/en/archive-documents/budget-speech-17.html?lang=. Published July 2014 |
| 29. | Ministry of Finance. Budget Speech of Fiscal Year 2020/21. https://mof.gov.np/en/archive-documents/budget-speech-17.html?lang=. Published May 2020 |
| 30. | Ministry of Health and Population. Financial Management Improvement Plan 2016-2021. http://www.nhssp.org.np/NHSSP_Archives/health_financing/FMIP_2016_2021_January2017.pdf. Published January 2017 |
| 31. | Ministry of Health and Population. Nepal Health Sector Strategy Implementation Plan 2016-2021. https://climate.mohp.gov.np/downloads/Nepal_Health_Sector_Strategy_Implementation_Plan_2016-2021.pdf. Published 2017 |
| 32. | Ministry of Health and Population, World Health Organisation. Multisectoral Action Plan for the Prevention and Control of Non Communicable Diseases (2014-2020). http://origin.searo.who.int/nepal/documents/noncommunicable_diseases/ncd_multisectoral_action_plan.pdf. Published 2014 |
| 33. | Gautam RP, Prasain JN. Current Situation of Occupational Safety and Health in Nepal. https://gefont.org/assets/upload/downloads/Study_OSH_Nepal.pdf. Published 2011 |
| 34. | Ministry of Health and Population, NHSSP. A Concept Note Transaction Accounting and Budget Control System (TABUCS). http://www.nhssp.org.np/NHSSP_Archives/health_financing/TABUCS_concept_july2011.pdf. Published July 2011 |
| 35. | World Health Organization. Global status report on noncommunicable diseases 2010. https://www.who.int/nmh/publications/ncd_report2010/en/. Published 2011 |
| 36. | Nepal Health Research Council (NHRC), Ministry of Health and Population (MoHP), Monitoring Evaluation and Operational Research (MEOR). Nepal Burden of Disease 2017: A Country Report based on the Global Burden of Disease 2017 Study. http://nhrc.gov.np/wp-content/uploads/2019/04/NBoD-2017_NHRC-MoHP.pdf. Published April 2019 |
| 37. | Department of Health Services. Annual Report Department of Health Services 2074/75 (2017/18). https://dohs.gov.np/annual-report-2074-75/. Published July 2019 |
| 38. | Department of Health Services. Health Management Information System Guidelines 2017. https://dohs.gov.np/hmis-guideline-2017/. Published March 2014. Updated May 2018 |
| 39. | Central Bureau of Statistics. National Population and Housing Census 2011 (National Report). https://unstats.un.org/unsd/demographic-social/census/documents/Nepal/Nepal-Census-2011-Vol1.pdf. Published November 2012 |
| 40. | Dhimal M, Bista B, Bhattarai S, Dixit LP, et al. Noncommunicable Disease Risk Factors: STEPS Survey Nepal 2019. https://www.who.int/docs/default-source/nepal-documents/ncds/ncd-steps-survey-2019-compressed.pdf?sfvrsn=807bc4c6_2. Published January 2020 |
| 41. | Nepal Health Sector Support Programme (NHSSP). Framework For Improved Management of Health Information In The Context Of Federal Governance Structures In Nepal. Kathmandu: https://www.nhssp.org.np/Resources/EA/Framework_for_Improved_Health_MIS_June2017.pdf. Published June 2017 |
| 42. | Kombe G, Lisa F, Eddie K, et al. Nigeria Health System Assessment 2008. https://www.hfgproject.org/nigeria-health-system-assessment-2008/. Published April 2009. |
| 43. | Mulaki A, S. Muchiri S. Kenya Health System Assessment. http://www.healthpolicyplus.com/ns/pubs/11328-11600_KenyaHSAReport.pdf. Published May 2019 |
| 44. | World Health Organization. Monitoring, evaluation and review of the national health strategies. https://www.who.int/classifications/ME_component_nationalhealthplans_prepub_july2011.pdf. Published 2011 |
| 45. | Centers for Disease Control and Prevention. CDC Kenya Annual Report 2018. https://www.cdc.gov/globalhealth/countries/kenya/reports/index.html. Published 2018 |
| 46. | The Nepal NCDI Poverty Commission. The Nepal NCDI Poverty Commission An Equity Initiative to Address Noncommunicable Diseases and Injuries National Report 2018. http://www.ncdipoverty.org/nepal-report. Published March 2018 |
| 47. | Department of Health Services. Annual Report DOHS 2071/72 (2014/15). https://dohs.gov.np/wp-content/uploads/2016/06/Annual_Report_FY_2071_72.pdf. Published May 2016 |
| 48. | Department of Health Services. Annual Report DOHS 2072/73 (2015/16). https://dohs.gov.np/wp-content/uploads/2017/06/DoHS_Annual_Report_2072_73.pdf. Published February 2017 |
| 49. | Khadka K, Bhattarai C. Source book for 21 Social Accountability Tools. http://documents1.worldbank.org/curated/en/513571468059674130/pdf/718040WP00PUBL0ebook0English0Final0.pdf. Published February 2012 |
| 50. | World Bank. World Bank Annual Report 2017. http://pubdocs.worldbank.org/en/908481507403754670/Annual-Report-2017-WBG.pdf. Published 2017.doi: 10.1596/978- 1-4648-1119-7. |
| 51. | Ministry of Health and Population. Nepal Health Infrastructure Development Standards 2017. https://dohs.gov.np/nepal-health-infrastructure-development-standards-2017/. Published May 2017 |
| 52. | Aryal KK, Neupane S, Mehata S, et al. Non-Communicable Diseases Risk Factors: STEPS Survey 2013. http://nhrc.gov.np/wp-content/uploads/2017/02/noncommunicable-disease-report_2012_2013.pdf. Published 2014 |
| 53. | Carlos A, Bright R, Gutierrez J, Hoadley K, Manuel C, Romero N and Rodriguez M P.  Guatemala Health system Assessment. Bethesda, MD: Health Finance & Governance Project, Abt Associates Inc; 2015. https://www.usaid.gov/sites/default/files/documents/1862/Guatemala-HSA%20_ENG-FULL-REPORT-FINAL-APRIL-2016.pdf |
| 54. | Ministry of Health, GOvernment of Kenya. 2013 Kenya Household Health Expenditure And Utilisation Survey. Nairobi: Government of Kenya; 2014. https://www.healthpolicyproject.com/pubs/745_KHHUESReportJanuary.pdf |
| 55. | World Health Organization. Workforce requirements for universal health coverage and the sustainable development goals: Human Resources for Health Observer Series No 17. 2016. Geneva; 2016. https://apps.who.int/iris/bitstream/handle/10665/250330/9789241511407-eng.pdf;jsessionid=8083C261916CC2873054900058289A0E?sequence=1https://apps.who.int/iris/bitstream/handle/10665/250330/9789241511407-eng.pdf;jsessionid=06FF9C405C5E3CB27771D5CF379671AE?sequence=1 |
| 56. | Institute for Health Metrics and Evaluation (IHME). Findings from the Global Burden of Disease Study 2017. Seattle, WA: IHME; 2018. http://www.healthdata.org/sites/default/files/files/policy_report/2019/GBD_2017_Booklet_Issuu_2.pdf |
| 57. | Cardiac Society of Nepal (CSON). Total number of Cardiologists/ Cardiac Surgeons as of March 2019. Kathmandu: CSON; 2019 |
| 58. | Nepal Health Professional Council (NHPC). Total Registered Health Professional 2019.Kathmandu. NHPC 2019 |
| 59. | Nepal Medical Council (NMC).Total registered doctors (including specialized doctors) as of December 31, 2019.Kathmandu.NMC; 2019 |
| 60. | Nepal Nursing Council (NNC).Nurses and ANMS as of 22nd April,2019.Kathmandu:NNC; 2019 |
| 61. | Ministry of Health and Population. Procurement Statement FY 2074/75. http://dohslmd.gov.np/web/en/postdetail/procurement-statement-of-fy-2074-75. Published August 2018 |
| 62. | National Health Training Center (NHTC) . Package of Essential Non-Communicable Diseases(PEN) intervention at Primary Service Setting Training Trainer’s Guide. Kathmandu: Ministry of Health and Population; 2075 |
| 63. | Ministry of Health and Population (MoHP). Status of Health Workforce in Nepal: 14 HRH indicator monitoring report. National Health Workforce Registry Program, MoHP; 2019 |
| 64. | WorldBank. Nepal Healthcare Spending 2000-2020. macrotrends. Published online 2019. Accessed August 4, 2020. <https://www.macrotrends.net/countries/NPL/nepal/healthcare-spending> |
| 65. | World Health Organization, Global Health Workforce Alliance. A Universal Truth: No Health Without A Workforce. World Health Organization; 2014. https://www.who.int/workforcealliance/knowledge/resources/GHWA-a_universal_truth_report.pdf?ua=1. |
| 66. | Non Communicable Diseases and Mental Health Section, Epidemiology and Disease Control Division (EDCD). Budget for NCD and Mental Health 2019/20. Presented at the: National Annual Review Meeting; 2019; EDCD, Department of Health Services (DoHS). |
|  |  |
| **Websites:** | |
| 1. | Department of Drug Administration. Pharmacovigilance. http://www.dda.gov.np/content/pharmacovigilance. |
| 2. | Department of Drug Administration.Role of DDA. <http://www.dda.gov.np/content/role-of-dda> |
| 3. | Public Procurement Monitoring Office. About PPMO. https://ppmo.gov.np/about_us/about_ppmo |
| 4. | World Population Review.Nepal Population 2020 (Live). https://worldpopulationreview.com/countries/nepal-population Accessed 8 January 2020. |
| 5. | World Food Programme,Logistic Capacity Assessment. Nepal Storage Assessment: Cold Chain  https://dlca.logcluster.org/display/public/DLCA/2.6+Nepal+Storage+Assessment |
| 6. | Government of Nepal. Salary of Civil Servants (Government Officials) in Nepal. https://edusanjal.com/blog/salary-civil-servants-nepal/. Published July 2019. |
| 7. | Pharmacy at a glance. Nepal Pharmacy Council. Pharmacy at a glance, https://www.nepalpharmacycouncil.org.np/post/pharmacy-at-a-glance. Accessed June 5, 2020. |
| 8. | Health Insurance board. Health Insurance latest information, Health Insurance Dashboard.  http://dashboard.hib.gov.np:81/. Accessed June 6, 2020 |
| 9. | Nepal: HIS Indicators - MEASURE Evaluation. Nepal: HIS Indicators. https://www.measureevaluation.org/his-strengthening-resource-center/country-profiles/nepal. Published October 30, 2019. |
| 10. | Afghanistan: HIS Indicators. MEASURE Evaluation. Afghanistan: HIS Indicators https://www.measureevaluation.org/his-strengthening-resource-center/country-profiles/afghanistan. Published October 30, 2019. |
| 11. | Ghana: HIS Indicators. MEASURE Evaluation. Ghana: HIS Indicators. https://www.measureevaluation.org/his-strengthening-resource-center/country-profiles/ghana . Published October 30, 2019 |
| 12. | Bangladesh: HIS Indicators. MEASURE Evaluation. Bangladesh: HIS Indicators. https://www.measureevaluation.org/his-strengthening-resource-center/country-profiles/bangladesh . Published October 30, 2019 |
| 13. | Ethiopia: HIS Indicators. MEASURE Evaluation. Ethiopia: HIS Indicators. https://www.measureevaluation.org/his-strengthening-resource-center/country-profiles/ethiopia. Published October 30, 2019 |
| 14. | Central Bureau of Statistics. Total Population. Government of Nepal.2017.https://cbs.gov.np/population-2011/ |
| 15. | Central Bureau of Statistics. Total Population. Government of Nepal. 2018.https://www.mope.gov.np/downloadfile/National%20Population%20Report%202017_1515491881.pdf |
| 16. | Nepal Health Facility Registry, Dec, 2019  National Health Facility Registry .MOHP. National Health Facility Registry. https://nhfr.mohp.gov.np/.Accessed December,2019 |
| 17. | Rural Health care system in India. Government of India. Rural Healthcare System in India  https://main.mohfw.gov.in/sites/default/files/Final RHS 2018-19_0.pdf . Published 2017. |
| 18. | World Bank. Nurses and midwives (per 1,000 people) - Nepal. https://data.worldbank.org/indicator/SH.MED.NUMW.P3?locations=NP&name_desc=false. Accessed August 4, 2020. |
| 19. | World Bank. Physicians (per 1,000 people) - Nepal. https://data.worldbank.org/indicator/SH.MED.PHYS.ZS?locations=NP&name_desc=false. Accessed August 4, 2020. |
| 20. | World Health Organization. Fact sheets: Non-Communicable Diseases. World Health Organization. https://www.who.int/en/news-room/fact-sheets/detail/cardiovascular-diseases-(cods). Published January 2015 |
| 21. | Trust Jayanti Memorial. About Jayanti Memorial Trust. http://www.jmt.org.np/about-us . Accessed July 30, 2020 |

**Supplement B.**

**List of Cardiac care related services provided by Health Insurance**

| **S.N.** | **Services** |
| --- | --- |
| 1 | Aortic Valve Balloon Dilation (AVBD) (Balloon Extra) |
| 2 | Atrial Septal Defect (ASD) Device Closure |
| 3 | Balloon Septostomy (Balloon Extra) (with contrast) |
| 4 | Patent Ductus Arteriosus (PDA) Device Closure |
| 5 | Pericardiocentesis (Except Aspiration Catheter) |
| 6 | Pulmonary Artery Thrombectomy / Embolectomy (with catheter) |
| 7 | Ventricular Septal Defect (VSD) Device Closure (with contrast) |
| 8 | Permanent Pacemaker Implantation (Pacemaker Generator Extra) |
| 9 | Epicardial Pacemaker Insertion |
| 10 | Single Pacemaker insertion |
| 11 | Dual Pacemaker insertion |
| 12 | Programming Single Pacemaker |
| 13 | Programming Dual Pacemaker |
| 14 | 14 External C Pap Care |
| 15 | EP Study |
| 16 | EP/Ablation Type A (with contrast) |
| 17 | EP/Ablation Type B (with contrast) |
| 18 | ABP Monitoring |
| 19 | Ambulatory BP Monitoring (ABPM) |
| 20 | Ankle Brachial Index (ABI) |
| 21 | Bilateral Limb Arterial Doppler |
| 22 | Bilateral Limbs Venous Doppler |
| 23 | Carotid Doppler |
| 24 | External C pap Care For 24 Hours |
| 25 | Holter Monitoring |
| 26 | Insertion Of Arterial Line |
| 27 | Insertion Of Swan gauge Catheterization |
| 28 | Left Heart Catheterization Including Contrast |
| 29 | Renal Doppler |
| 30 | Right Heart Catheterization (Oxymetry Extra) with contrast |
| 31 | Single Limb Arterial Doppler |
| 32 | Single Limb Venous Doppler |
| 33 | Stress ECHO |
| 34 | TEE (Transesophageal Echocardiography) |
| 35 | Tilting Table Test |
| 36 | Umbilical Artery Doppler |

| **Health center** | **Services available** |
| --- | --- |
| Shahid Gangalal National Heart Center (SGNHC) | 1. Anesthesia for open heart surgery 2. OPD services: Consultation, Echo, Fetal Echo, Carotid Doppler Echo, Stress Echo, ECG, TMT, Holter, ABP, X-ray 3. IPD services: OT, Surgical ICU, Medical ICU, Coronary care unit, General ward, Surgical ward, Cabin, Cathlab, pre-catch, electrophysiology studies 4. Cardiac Rehabilitation and Health Promotion: Consultation, Echo, fetal Echo, Caratoid Doppler Echo, Stress Echo, ECG, TMT, Holter, ABP, X-ray |
| Manmohan Cardiothoracic Vascular and Transplant Center (MCVTC) | a. Cath lab  b. Radiology  c. The Cardiothoracic and Vascular Anesthesia (CTVA)  d.Cardiothoracic and vascular surgery  e. Cardiology: Electrocardiograph, Echocardiography, Coronary care unit, Cathlab with cardiac intervention procedures, closed heart surgery and open heart surgery for ASD closures, simple cardiac intervention procedures like pacemaker implantation.  f. Non-invasive cardiac lab |
| District Hospitals | -No infrastructure and capacity for cardiac surgery and interventional cardiology  -Cardiac monitor, ECG and cardiac medicines are available |

Number of cath labs in different provinces of Nepal

| Province | Number of cath lab |
| --- | --- |
| Province 1 | 4 |
| Province 3 | 14 |
| Province 4 | 1 |
| Province 5 | 2 |
| Province 2,6 & 7 | 0 |
| Total | 21 |
|  |  |

Related Health Services available at PEN implemented facilities

| **Health Facilities** | **Services** | **Basic Diagnostics** |
| --- | --- | --- |
| Health Post | Detect hypertension, diabetes, assess CVD risks, counseling, referral, follow up of low risk patients, refill drugs, health promotion | Blood pressure measurement device, Glucometer, Urine protein test strips, Urine ketone test strips, Stethoscope, Measurement tape, Digital weighing scale, Stadiometer, CVD risk charts |
| Primary Health Care Centers | Confirm diagnosis of diabetes, hypertension, manage, follow up high risk patients, refer complicated cases to district hospital | Blood pressure measurement device, Glucometer, Cardio check, Urine protein test strips, Urine ketone test strips, Stethoscope, CVD risk charts, Measurement tape, Digital weighing scale, Stadiometer |
